# Nitrocellulose-bound achromopeptidase for point-of-care nucleic acid tests

**DOI:** 10.1038/s41598-021-85481-2

**Published:** 2021-03-17

**Authors:** Georgios Chondrogiannis, Shirin Khaliliazar, Anna Toldrà, Pedro Réu, Mahiar M. Hamedi

**Affiliations:** School of Engineering Sciences in Chemistry, Biotechnology and Health, KTH Royal Institute of Technology, Stockholm, Sweden

**Keywords:** Proteases, Diagnosis, Immobilized enzymes

## Abstract

Enzymes are the cornerstone of modern biotechnology. Achromopeptidase (ACP) is a well-known enzyme that hydrolyzes a number of proteins, notably proteins on the surface of Gram-positive bacteria. It is therefore used for sample preparation in nucleic acid tests. However, ACP inhibits DNA amplification which makes its integration difficult. Heat is commonly used to inactivate ACP, but it can be challenging to integrate heating into point-of-care devices. Here, we use recombinase polymerase amplification (RPA) together with ACP, and show that when ACP is immobilized on nitrocellulose paper, it retains its enzymatic function and can easily and rapidly be activated using agitation. The nitrocellulose-bound ACP does, however, not leak into the solution, preventing the need for deactivation through heat or by other means. Nitrocellulose-bound ACP thus opens new possibilities for paper-based Point-of-Care (POC) devices.

## Introduction

Enzymes are catalytical proteins that constitute crucial tools in biotechnology^1–3^. One particular area that uses many enzymes is Nucleic Acid Amplification Tests (NAATs)^4^. These diagnostic tests are capable of target detection with high sensitivity and specificity^5^, using three steps: (i) sample preparation, (ii) DNA amplification, and (iii) DNA detection. Their main disadvantage however is the requirement of high-end equipment and highly trained personnel, for carrying out these steps, which limits their use in POC devices. To overcome these limitations, numerous NAAT systems have been developed for the POC^5–8^. Out of these efforts, paper-based microfluidic diagnostic systems, often termed µPADs^9^ are showing high potential to minimize costs and enable mass-production of POC NAATs. Contrary to PCR, RPA is particularly well suited for integration into POC devices since it is an isothermal DNA amplification method, and therefore, does not require a thermocycler^6,10^.

Even though a number of techniques have been presented for paper-based amplification and detection^5^, much less attention has been devoted to the development of sample preparation, wherein cell lysis and nuclear acid purification must occur prior to amplification. The purification steps generally require the removal of all compounds present in the lysate, including the lysis reagents which may inhibit downstream processes such as DNA amplification and detection^5^.

To integrate sample preparation in µPAD NAATs, Whatman FTA™ paper, a proprietary material used to extract DNA from cells and preserve it at room temperature, has been used^11,12^. FTA™ paper, however, introduces amplification inhibitors and requires a series of washing steps which makes its integration into POC diagnostics difficult^11,13–15^. This is an inherent limitation that occurs when utilizing chemicals for lysis, which denature proteins nonspecifically and cannot be deactivated.

Enzymes in solution can also inhibit or otherwise negatively affect downstream reactions, and therefore have to be deactivated. Peptidases form a subgroup of enzymes that catalyze the hydrolysis of proteins^16^. Peptidases such as proteinase K, papain and ACP are commonly used to digest tissues and cells^17–19^. ACP is a mixture of enzymes known to efficiently lyse Gram-positive bacteria and that has been used in POC systems^20–22^. In order to proceed with amplification or other downstream steps ACP must, however, first be deactivated. ACP deactivation is typically achieved by heat^5,20–22^. The same is true in the cases of lambda exonuclease^6,8^ and DNase I^23^. For integration into POC NAATs, this introduces complexity since the minimum temperature required for ACP deactivation is 80 °C^20^.

Therefore, there is a need to develop methods for NAAT sample preparation that allows the utilization of enzymes such as ACP, but omits the need for downstream heat deactivation or multiple washing steps. Here, we use nitrocellulose paper to immobilize ACP and enable its utilization preventing it from entering downstream solutions, eliminating the need to deactivate it.

## Results and discussion

### Concept

**Figure 1:**
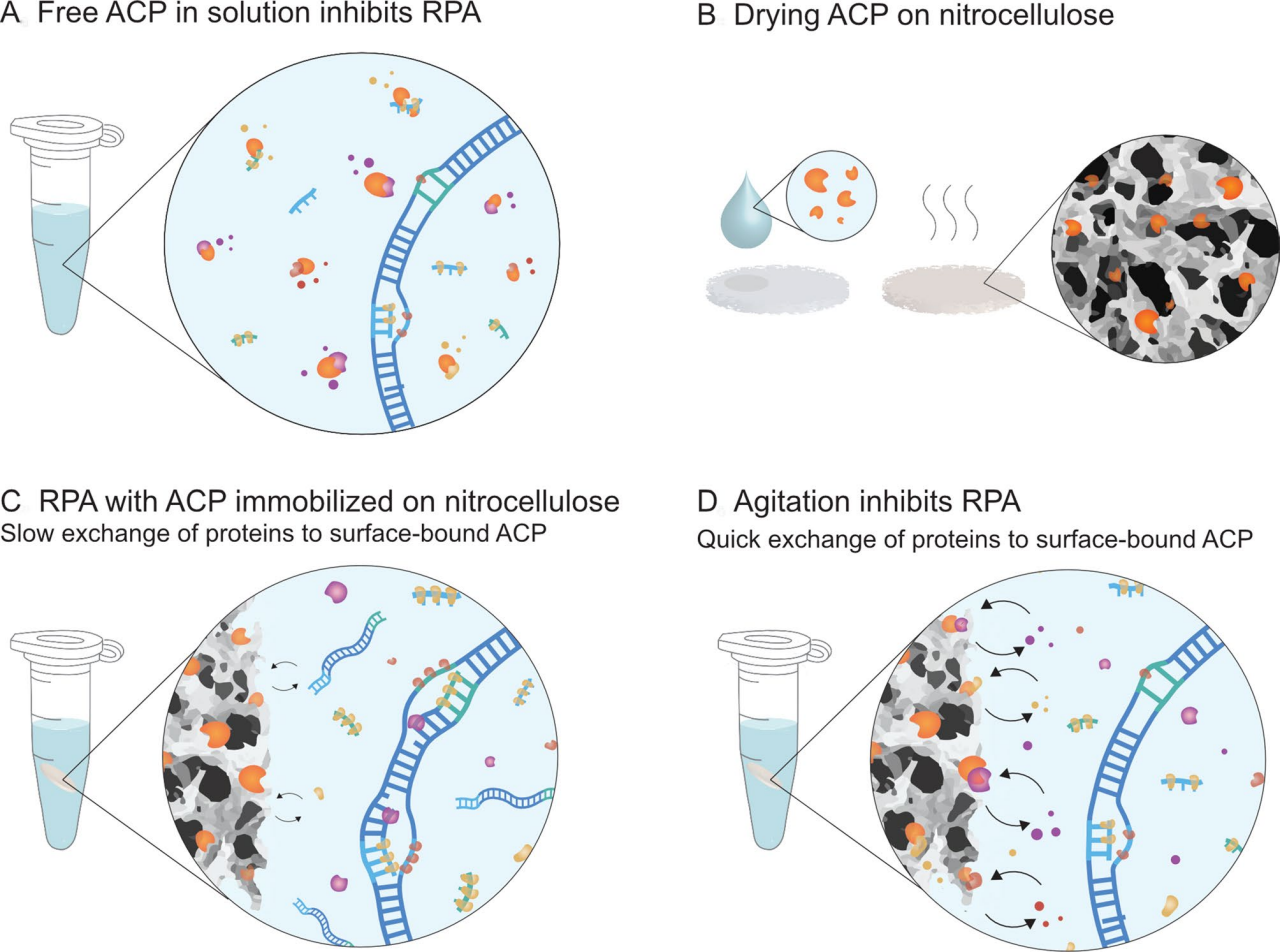
Schematic representation of the concepts. (**A**) Free ACP in solution inhibits RPA possibly by digesting the enzymes necessary for amplification; (**B**) drying ACP on nitrocellulose for immobilization. (**C**) Undisturbed ACP immobilized on nitrocellulose does not immediately inhibit RPA. (**D**) Agitation probably increases the rate at which RPA reagents come in contact with active ACP on the surface of nitrocellulose.

ACP has been extensively used for sample preparation given its efficiency in lysing bacteria namely *S. epidermidis*, a gram-positive bacterium which can be particularly hard to lyse due to its cell wall^19,24–26^. ACP is known to inhibit downstream reactions^20,27^ (Fig. 1A), hence the use of ACP in NAATs requires additional steps after sample preparation such as high temperature to deactivate the enzymes^5,21,28^. Here, we set out to investigate if drying ACP to nitrocellulose (Fig. 1B) could yield a functional yet immobilized ACP. We used RPA to assess the activity of nitrocellulose-bound ACP both when undisturbed (Fig. 1C) and when agitated (Fig. 1D). Nitrocellulose paper is a widely available material used for instance in Western Blot analysis^29^, in paper-based immunoassays^6,30^, and in paper-chips for nucleic acid amplification^31^. Nitrocellulose is well suited for enzyme-based sample preparation, given its capacity to bind proteins, a process well established since the 1960s^32,33^.

### Nitrocellulose-bound ACP

**Figure 2:**
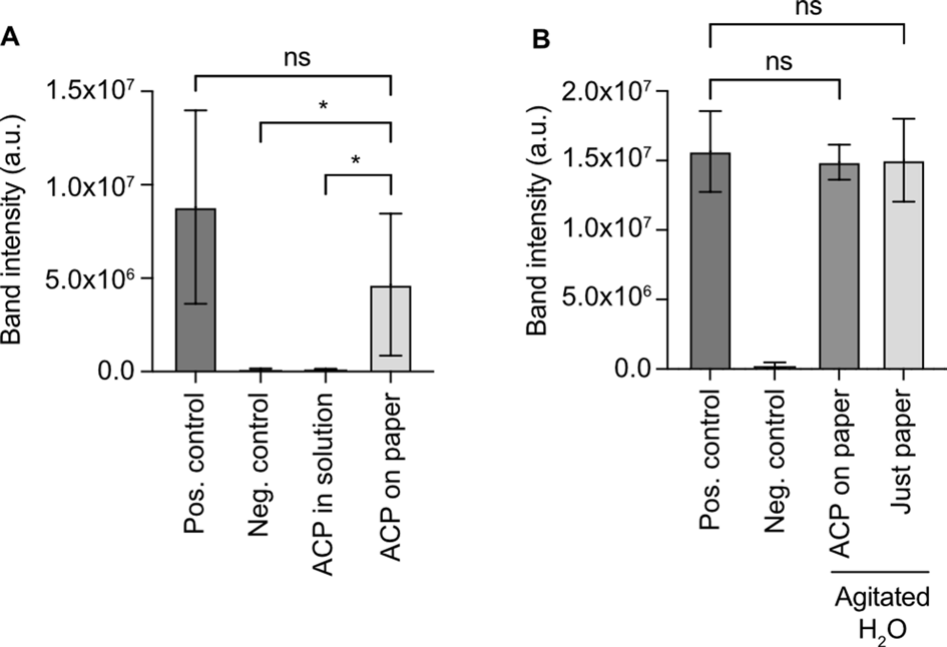
Nitrocellulose-bound ACP. (**A**) Densitometric analysis of gel electrophoresis results of Fig. S1. While ACP in solution inhibits RPA completely, undisturbed nitrocellulose-bound ACP does not significantly affect RPA (n = 5 for all conditions, unpaired t-test, mean with SD). (**B**) Densitometric analysis of gel electrophoresis results of Fig. S2. Agitation of nitrocellulose-bound ACP in water did not release enough ACP to significantly inhibit RPA (after the removal of the nitrocellulose, the water was used in the RPA). Furthermore, water in which plain nitrocellulose was agitated, does not significantly inhibit RPA either (n = 5 for all conditions, unpaired t-test, mean with SD).

We added 1 μl of ACP on a two-millimeter (in diameter) nitrocellulose disc and dried at 37 °C for 15 min to ensure complete drying. This temperature is often chosen when using ACP. We tested the effect of free ACP in solution and nitrocellulose-bound ACP on RPA by performing amplification of genomic DNA extracted from *S. epidermidis* (Fig. S1). We ran the amplification products from all the experiments in the same electrophoresis gel for quantification purposes (Fig. S1). According to the densitometric analysis, the intensity of target band (210 bp) in the presence of nitrocellulose-bound ACP was not significantly different from the positive control (Fig. 2A). Furthermore, the target band intensity was significantly higher than that of the negative control and RPA with free ACP in solution (Fig. 2A). This demonstrates that, contrary to ACP in solution, nitrocellulose-bound ACP does not have an immediate inhibitory effect on RPA.

### Effect of diffusion and agitation on the activity of nitrocellulose-bound ACP

To evaluate the bond between the ACP and the nitrocellulose we put nitrocellulose-bound ACP in water and agitated it (Fig. S2). This water, without the nitrocellulose, was used in an RPA reaction and did not affect it, demonstrating that ACP remains largely immobilized on the nitrocellulose despite thorough agitation (Fig. 2B). Furthermore, we showed that water, in which plain nitrocellulose had been agitated, does not present measurable inhibitory effects on RPA either (Figs. 2B, S2).

**Figure 3:**
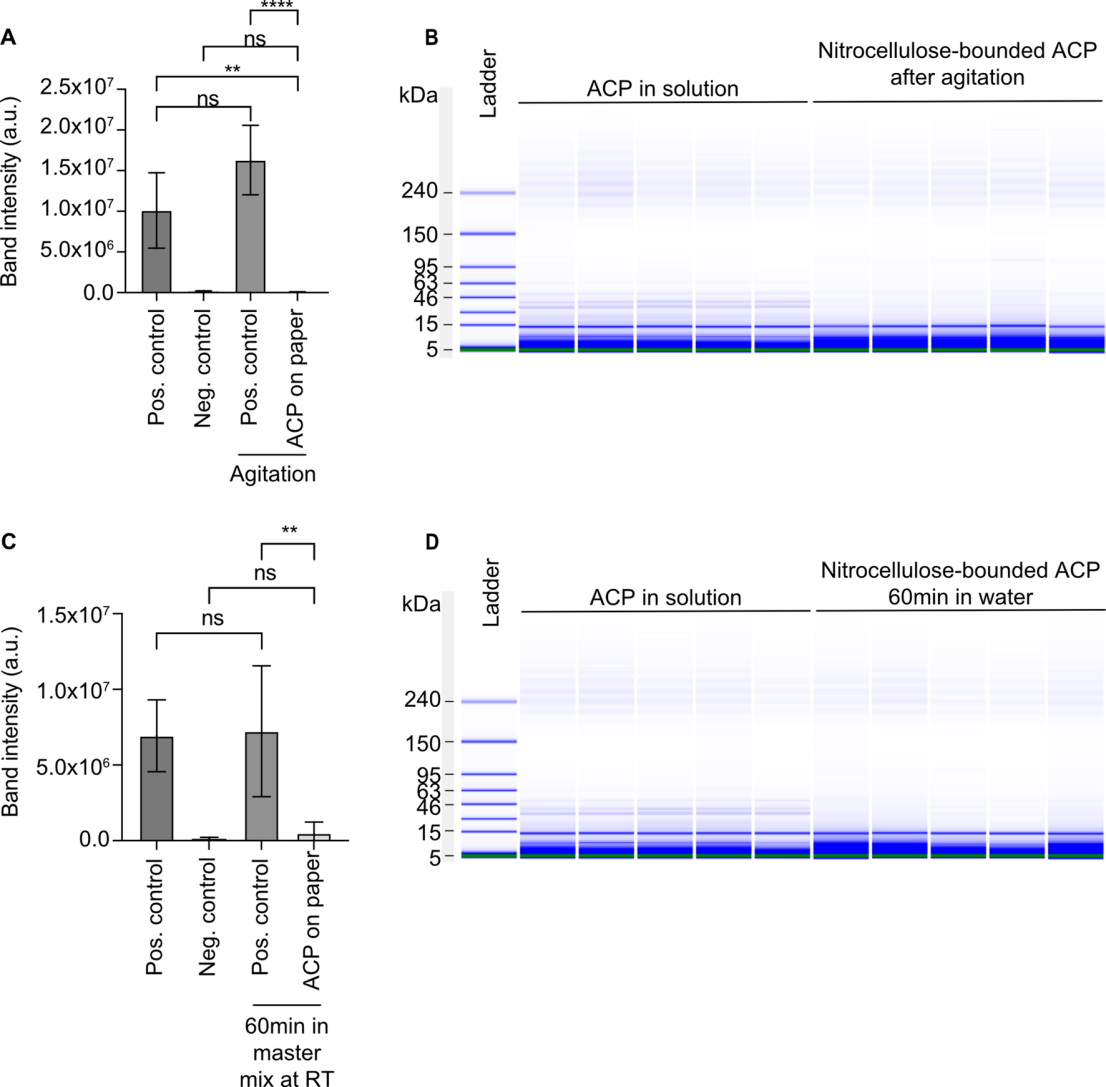
Effect of diffusion and agitation on the activity of nitrocellulose-bound ACP. (**A**) Densitometric analysis of agarose gel electrophoresis results in Fig. S3 showing that nitrocellulose-bound ACP is capable of RPA inhibition when mixed (n = 5 for all conditions, unpaired t-test, mean with SD). (**B**) No ACP (30 to 50 kDa) was detected by chip-based electrophoresis in water where nitrocellulouse-bound ACP was submitted to agitation. Full-length gel image is presented in Fig. S5. (**C**) Densitometric analysis of agarose gel electrophoresis results in Fig. S6 showing that nitrocellulose-bound ACP is capable of high to complete RPA inhibition within 60 min, possibly by diffusion of amplification enzymes to the ACP on the paper surface (n = 5 for all conditions, unpaired t-test, mean with SD). (**D**) No ACP (30 to 50 kDa) was detected by chip-based electrophoresis in water where nitrocellulose-bound ACP was left for 60 min. Full-length gel image is shown in Fig. S7.

To test whether nitrocellulose-bound ACP activity is affected by mixing, we performed RPA following agitation of the RPA mix containing nitrocellulose-bound ACP (Fig. S3). We analyzed the amplification products from all experiments in the same electrophoresis gel to allow for quantification (Fig. S3). Agitation did not significantly change band intensity for the positive control (Fig. 3A). It did however, result in a highly significant reduction in the presence of nitrocellulose-bound ACP (Fig. 3A). In fact, the intensity for nitrocellulose-bound ACP band was not significantly different from that of the negative control (Fig. 3A). In contrast, the presence and agitation of plain nitrocellulose in RPA mixture did not inhibit RPA (Fig. S4A,B).

To assess whether ACP could be released from the paper during agitation, we utilized chip-based capillary electrophoresis to compare ACP in solution with the ACP content in water following the agitation of nitrocellulose-bound ACP (Figs. 3B, S5). No release of ACP (30 to 50 kDa) was detected following agitation (Fig. 3B).

Furthermore, we investigated the activity of immobilized ACP on a reaction under passive diffusion by incubating nitrocellulose-bound ACP in RPA mix for 60 min (undisturbed) before initiating the RPA reaction (Figs. 3C, S6). The significant inhibition of RPA (Fig. 3C) can be plausibly explained by the diffusion of active RPA reagents to the surface of nitrocellulose where they reacted with the immobilized, yet active, ACP.

To assess the stability of nitrocellulose-ACP bond, we placed the nitrocellulose paper in water for 60 min, after which we analyzed it for traces of ACP by chip-based capillary electrophoresis (Figs. 3D, S7). Similarly, to the results shown in Fig. 3B, we did not detect any ACP with this method (Fig. 3D).

Nitrocellulose-bound ACP appeared stable when examined by chip-based capillary electrophoresis (Fig. 3B,D). The absence of bands from these samples compared to free ACP in solution suggests that ACP does not easily detach from the nitrocellulose (Fig. 3B,D). The same conclusion is supported by the fact that water, in which nitrocellulose-bound ACP had been thoroughly agitated, failed to affect the RPA reaction (Fig. 2B).

It seems that ACP can be stored in nitrocellulose without significantly affecting its function (Fig. 3A,C) and importantly, it can be used while immobilized (Fig. 3A,C), which allows for its removal from solution (Figs. 2B, 3B,D) without the need for heat or other deactivation. This is of particular relevance for the integration of enzymatic systems in portable devices where it is advantageous to have protocols with few and simple steps without external instrumentation^1,34^.

## Conclusion

We demonstrated that nitrocellulose-bound ACP does not immediately inhibit RPA amplification in a stationary solution, contrary to ACP in solution. Nitrocellulose-bound ACP can, however, be activated by agitation in solution without being released. The described mechanism opens the possibility to utilize nitrocellulose-bound ACP for reactions such as cell lysis in paper-based NAATs, or possibly for reaction inhibition in other applications. Once the bound ACP has carried out its function it can easily be removed from the solution without the need for instruments and without using heat deactivation or other processes that might affect downstream applications. We believe that this article paves the way for the improvement of POC devices by facilitating the integration of e.g., an instrument-free lysis step.

## Materials and methods

### Bacterial cultures and DNA extraction

A single colony of *Staphylococcus epidermidis* (ATCC 12228) was transferred from petri dishes with Difco nutrient agar (213000, Becton, Dickinson and Company, MD, USA) and cultured overnight in Difco nutrient broth (234000, Becton, Dickinson and Company) at 37 °C with shaking. The cells were spun at 14,000×*g* for 10 min and the pellet was used for DNA extraction with PureLink Microbiome DNA Purification Kit (Invitrogen, CA, USA) following the manufacturer’s instructions.

### Nitrocellulose and ACP preparation

1 μl of 30 U/μl ACP (A3547, Sigma Aldrich, MO, US) in Tris buffer (10 mM Tris, pH 8) was applied to a 2 mm nitrocellulose (10,600,003, GE Healthcare Life Science, Germany) disc. The disc was dried for 15 min at 37 °C. Once dried, the disc was carefully transferred into a 200 μl PCR tube containing the RPA mixture. To examine the effect of agitation, nitrocellulose containing ACP was agitated in 10 μl nuclease-free water by pipetting 50 times and stirred thoroughly using the pipette tip. This water was applied directly to the RPA mix.

### Primers

The primers for the SE-0105 gene of *S. epidermidis* were designed using Primer3 Output and IDT primer design tools, and were purchased from Biomers (Germany). The sequences of primers were TATAGGCTTAATTATCTCTGTTTTAGGAGCTT and TGATAGGCACTATCTGTAAACAA CATACTAAT for the forward and reverse primer respectively.

### DNA amplification

The extracted genomic DNA was amplified by RPA (TwistAmp Basic kit—TwistDx Ltd., Cambridge, UK). The RPA mix was prepared as shown on Tables 1 and 2.

**Table 1:** Volumes for each component present in RPA reactions for experiments in Fig. 2B.

Sample	Master Mix				Additional components		
	Rehydration buffer	H_2_O	Forward primer	Reverse primer	H_2_O	10^4^ copies of gDNA/μl	MgOAc
Positive control	29.5	2.2	2.4	2.4	10	1	2.5
Negative control	29.5	2.2	2.4	2.4	11	0	2.5
Nitrocellulose-bound ACP	29.5	2.2	2.4	2.4	10*	1	2.5
Nitrocellulose	29.5	2.2	2.4	2.4	10*	1	2.5

Volumes for each component present in RPA reactions in microliters.

*Water in which nitrocellulose containing ACP or plain was agitated.

**Table 2:** Volumes for each component present in RPA reactions for experiments in Fig. 3A,C, Fig. S4A,B. Volumes for each component present in RPA reactions in microliters.

Sample	Master Mix				Additional components			
	Rehydration buffer	H_2_O	Forward primer	Reverse primer	ACP	H_2_O	10^4^ copies of gDNA/μl	MgOAc
Positive control	29.5	11.2	2.4	2.4	0	1	1	2.5
Negative control	29.5	11.2	2.4	2.4	0	2	0	2.5
ACP in solution	29.5	11.2	2.4	2.4	1	0	1	2.5
Paper*	29.5	11.2	2.4	2.4	0	1	1	2.5

*Nitrocellulose paper plain for Fig. S4, or with dried ACP for Fig. 3A,C.

The master mix was used to rehydrate the RPA pellets which were mixed together in a tube and then redistributed to individual tubes. 2.5 μl of provided MgOAc solution was added to the lid of each tube. After a short centrifugation, the tubes were placed at 37 °C for 30 min. In the experiments where agitation was used to mix the nitrocellulose containing ACP or positive control, the tubes were inverted ten times and centrifuged again briefly prior to incubation at 37 °C for 30 min.

### Gel electrophoresis and band detection

The products of RPA reactions were purified using the QIAquick PCR Purification Kit (Qiagen, Germany) following the manufacturer’s instructions.

Agarose gel electrophoresis was used to examine the presence of DNA amplicons of the expected length after amplification. For each sample, 5 μl of amplification product was mixed with 1 μl of loading buffer (R1161, Thermo Scientific, MA, USA), and 5 μl of the mixture was ran in a 1.5% agarose (Agarose I, 0710, VWR, PA, US) gel with GelRed (41003—Biotium). DNA ladder (Generuler 50 bp, Thermo Fischer Scientific, MA, US) was used to estimate amplicon base pair length. The gel was imaged (Molecular Imager ChemiDoc XRS+—BioRad) and bands were quantified with Image Lab Software (BioRad).

Chip-based electrophoresis was performed in a Bioanalyzer (Agilent) and the samples were prepared with the Agilent High Sensitivity Protein kit (5067-1575) according to the manufacturer’s instructions.

## Supplementary Information

Supplementary Information.
