# Supplementary material for: Nitrocellulose-bound achromopeptidase for point-of-care nucleic acid tests
Source: Sci Rep. 2021 Mar 17;11:6140. doi: 10.1038/s41598-021-85481-2 (PMC7969615; doi:10.1038/s41598-021-85481-2)
Supplement: Supplementary file 1 — Supplementary Information. [file 41598_2021_85481_MOESM1_ESM.pdf]

Supplementary Information

for

Nitrocellulose-bound Achromopeptidase for Point-of-Care Nucleic Acid Tests

Georgios Chondrogiannis[1], Shirin Khaliliazar[1], Anna Toldrà[1], Pedro Réu[1#], Mahiar M. Hamedi[1#]

[1] School of Engineering Sciences in Chemistry, Biotechnology and Health, KTH Royal Institute of Technology, Stockholm Sweden

[#]Corresponding authors

Mahiar M. Hamedi (mahiar@kth.se)

Pedro Réu (pedrorc@kth.se)

Results

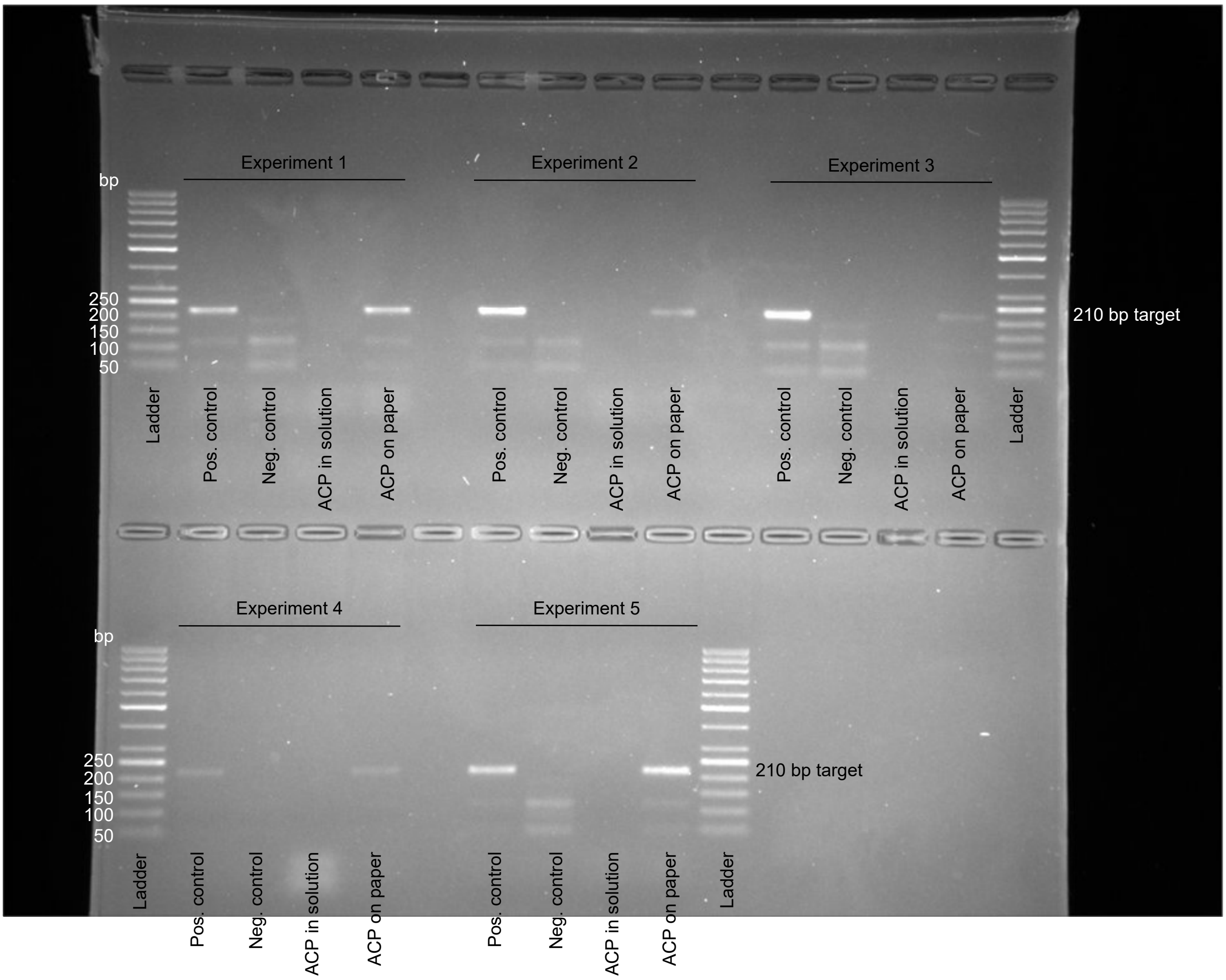

Figure S1. Full-length gel electrophoresis image showing the presence of 210bp band in the positive control and sample with nitrocellulose-bound ACP. Samples from five experiments were run in one gel for quantification purposes.

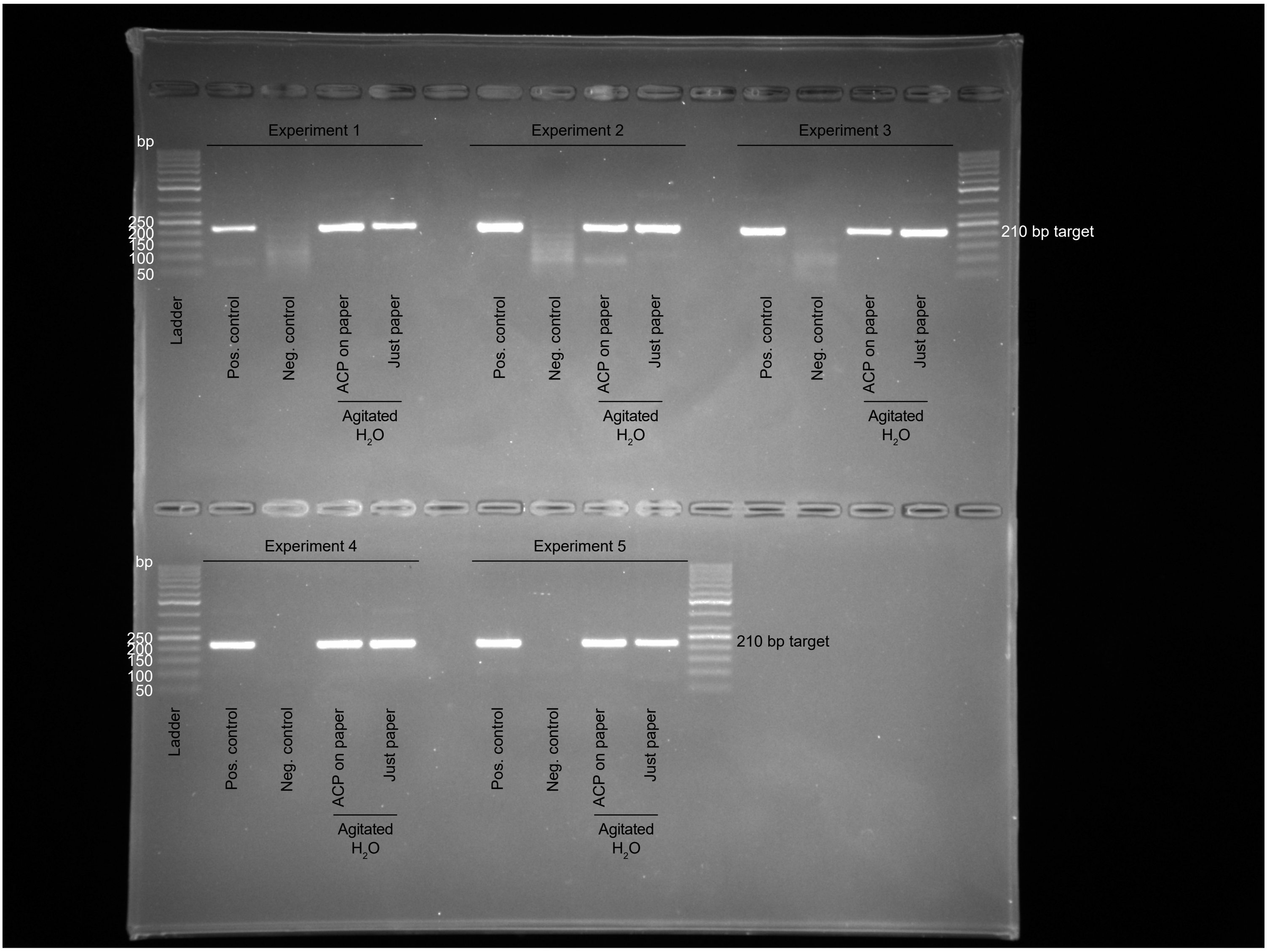

Figure S2. Full-length gel electrophoresis image with a 210 bp target band in the positive control and a similar result in the H_2O from the agitated ACP on paper and in the H_2O from the agitated control paper. Samples from five experiments were run in one gel for quantification purposes.

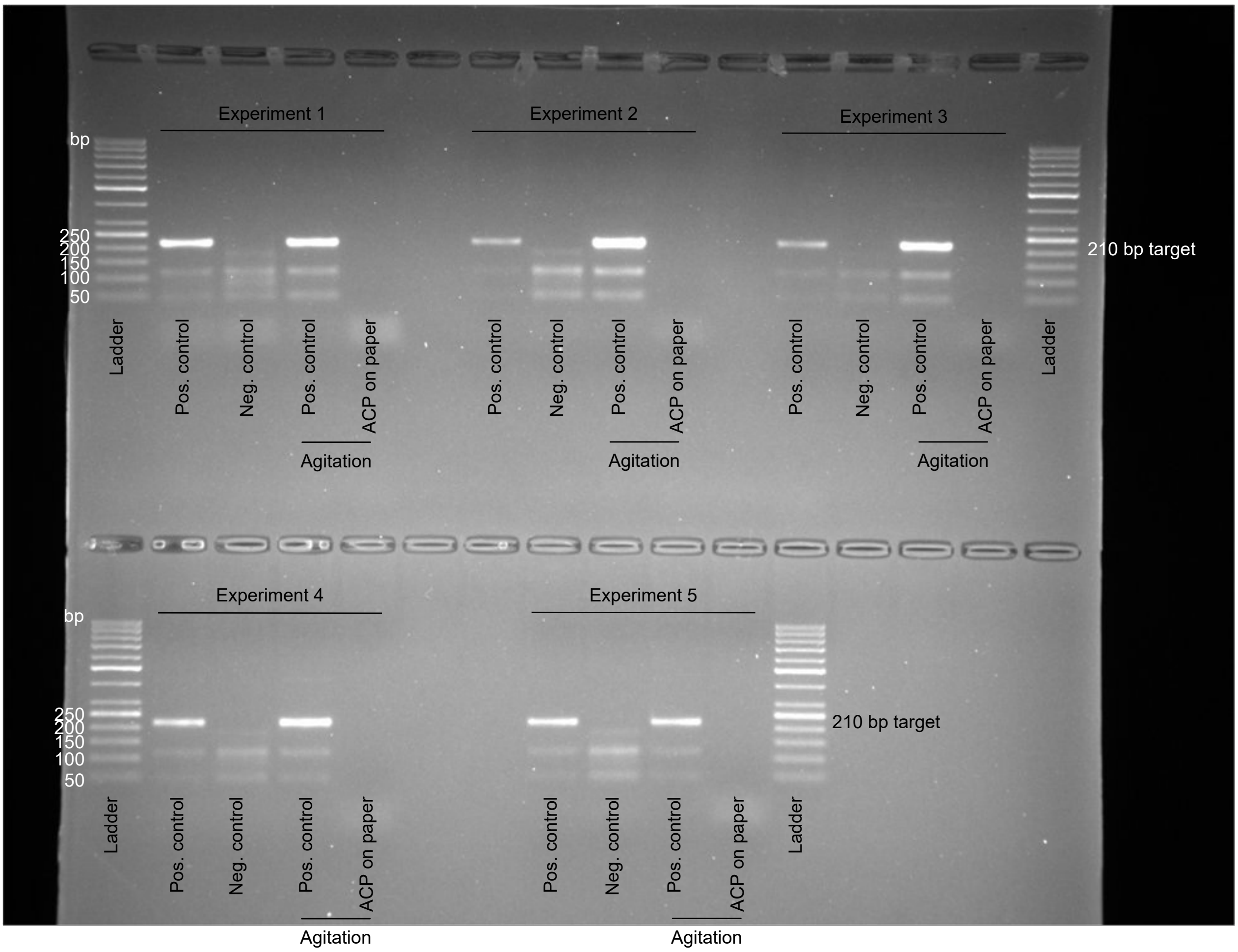

Figure S3. Full-length gel electrophoresis image with the 210 bp target band in the positive control and a similar result in the positive control with agitation. Agitation totally inhibited the reaction in the presence of ACP on paper. Samples from five experiments were run in one gel for quantification purposes.

A

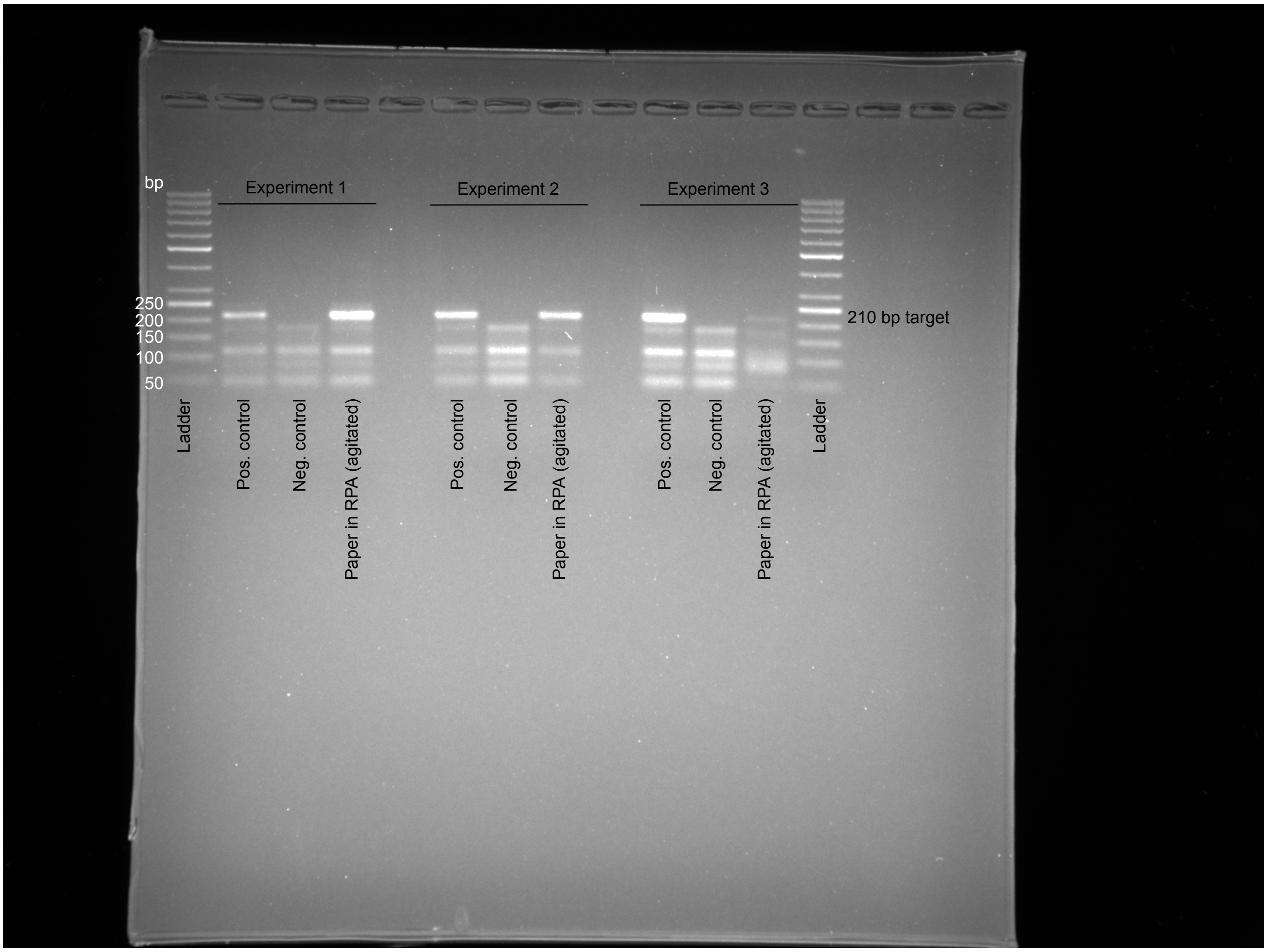

B

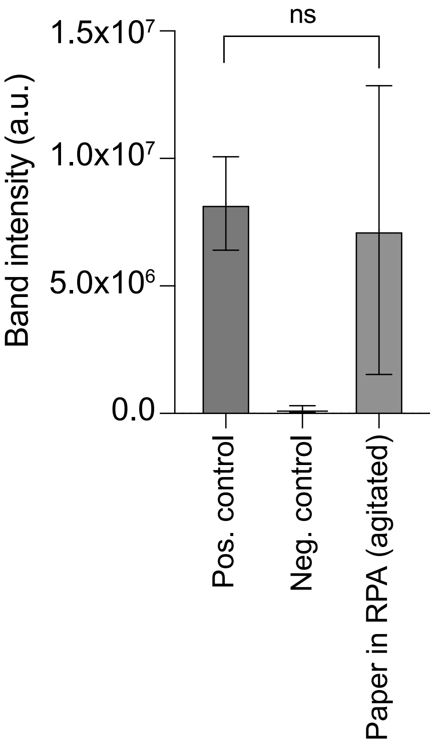

Figure S4. (A) Full-length gel electrophoresis image with a 210 bp target band in the positive control and a similar result in the sample with paper agitated inside the RPA mix. Samples from three experiments were run in one gel for quantification purposes. (B) Densitometric analysis of gel electrophoresis results. Plain nitrocellulose agitated inside the RPA mix prior to amplification does not seem to stop the RPA reaction (n=3 for all condi-tions, unpaired t-test, mean with SD).

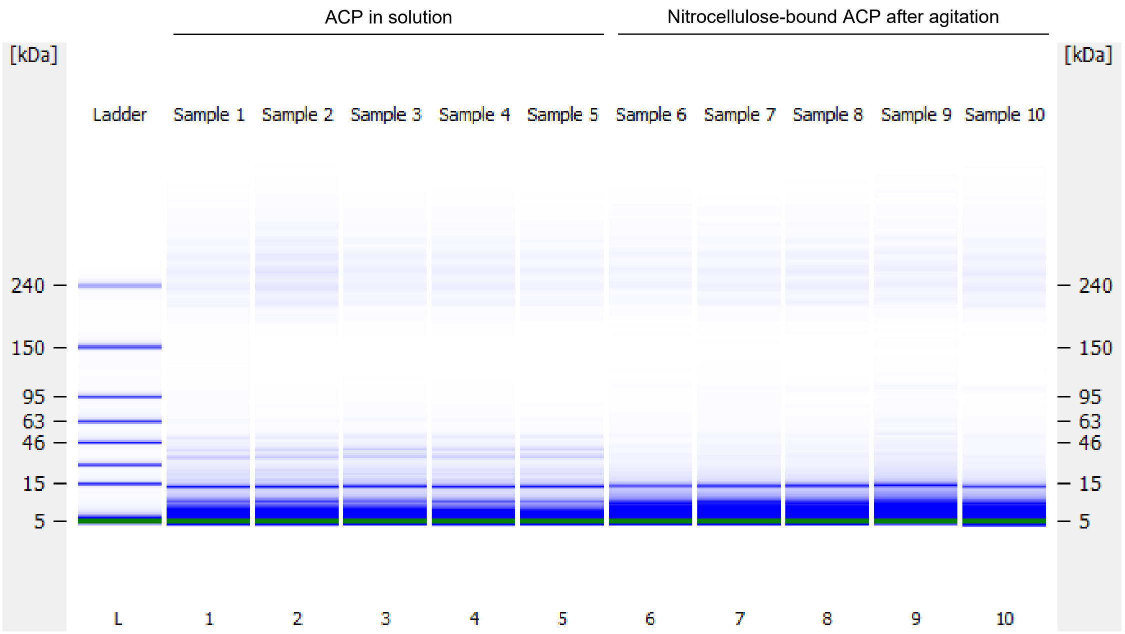

Figure S5. Full-length chip-based electrophoresis image from figure 3B, showing that agitation does not release ACP from the nitrocellulose.

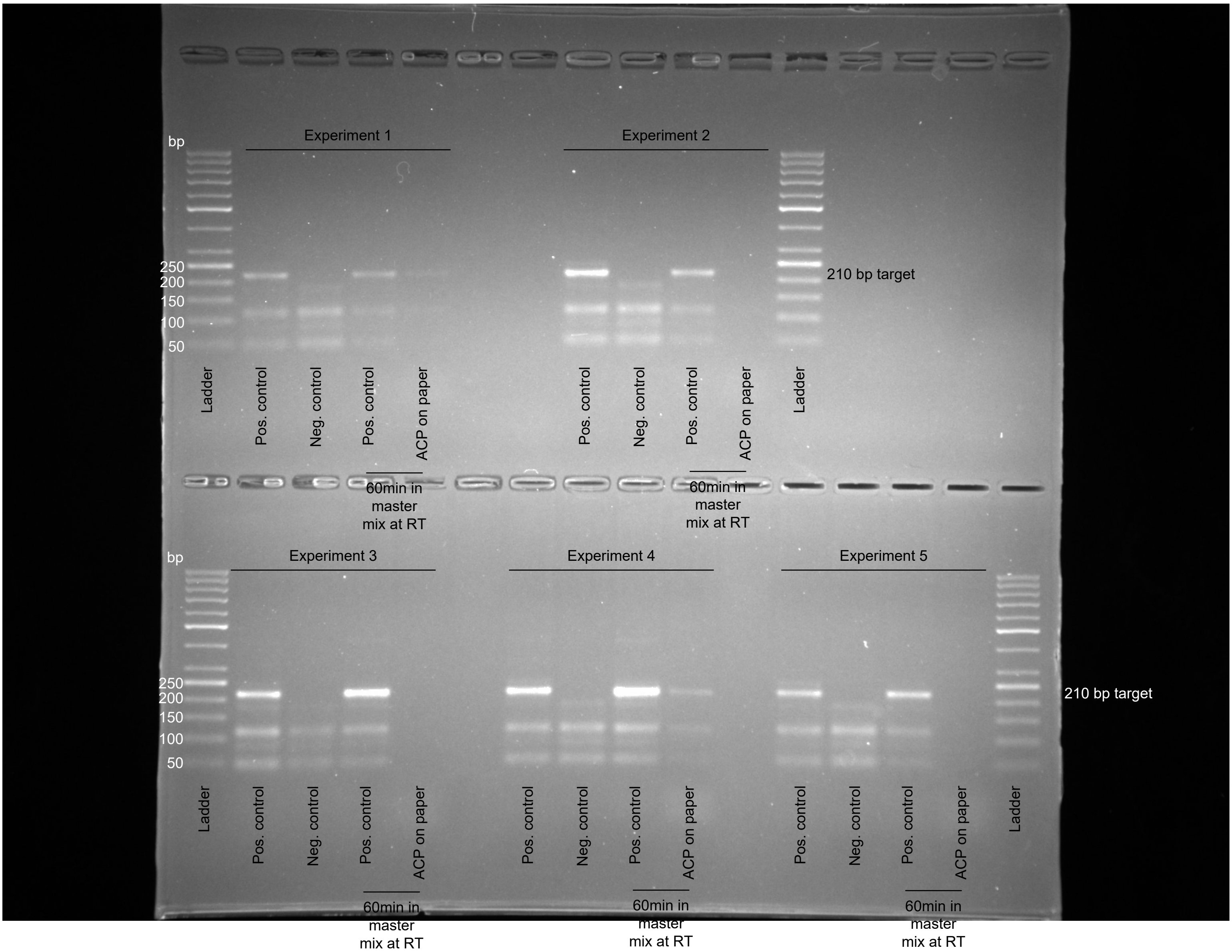

Figure S6. Full-length gel electrophoresis image with the 210 bp target band in the positive control and a similar result in the positive control after 60 min at room temperature. ACP on paper after 60 min at room temperature in RPA master mix highly to totally inhibits the subsequent reaction. Samples from five experiments were run in one gel for quantification purposes.

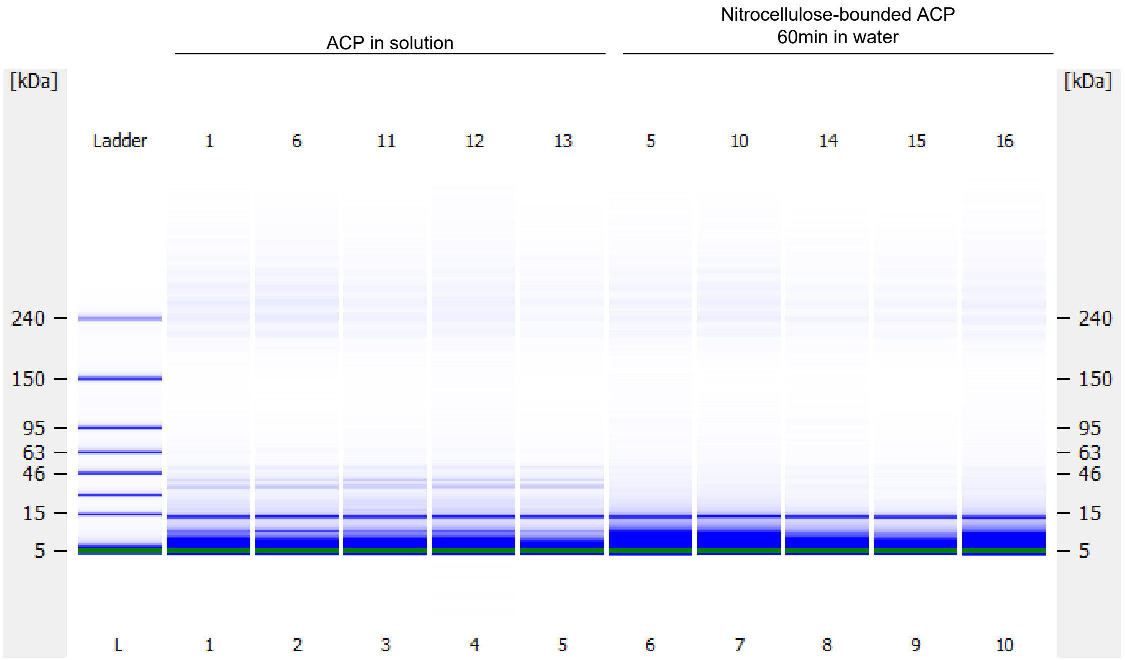

Figure S7. Full-length chip-electrophoresis image from figure 3D showing that ACP is not released from the nitrocellulose after 60 minutes in water.
